# Cost-effectiveness analysis of population-based *BRCA1/2* testing, family-history-based *BRCA1/2* testing, and symptom-based screening for breast and ovarian cancer in China

**DOI:** 10.3389/fpubh.2025.1479966

**Published:** 2025-06-11

**Authors:** Feilong Zhao, Shu Wang, Jianfei Lu, Xiangying Feng, Liwei Ran, Jianjun Yang

**Affiliations:** ^1^Department of Digestive Surgery, Xijing Hospital of Digestive Diseases, Fourth Military Medical University, Xi'an, China; ^2^State Key Laboratory of Holistic Integrative Management of Gastrointestinal Cancers and National Clinical Research Center for Digestive Diseases, Xijing Hospital of Digestive Diseases, Fourth Military Medical University, Xi'an, China; ^3^Institute for Hospital Management of Tsinghua University, Shenzhen, China; ^4^Department of Dermatology, Beijing Chao-Yang Hospital, Capital Medical University, Beijing, China

**Keywords:** BRCA1, BRCA2, ovarian cancer, breast cancer, family history, China, cost-effectiveness

## Abstract

**Background:**

The women’s cancer screening program has been operational for several years in China, primarily utilizing palpation and ultrasound. Given the proven impact of *BRCA1/2* mutations on the incidence of breast and ovarian cancer, the cost-effectiveness of incorporating *BRCA1/2* mutation testing into these programs, either for the entire population or through enrichment based on family history of breast and ovarian cancer, remains poorly researched.

**Methods:**

We constructed a decision tree model to compare the cost-effectiveness of three strategies: symptom-based screening only (Symptom-only strategy), population-based *BRCA1/2* testing (population-based strategy), and family-history-based *BRCA1/2* testing (FH-based strategy). One-way and probability sensitivity analyses enabled model uncertainty evaluation. Outcomes included early and advanced stages of ovarian and breast cancer. Cost, quality-adjusted life years (QALYs), and incremental cost-effectiveness ratios (ICERs) were calculated. The target population was women at 40–60 years, the time horizon was until age 70, and the perspective was payer-based.

**Results:**

The FH-based strategy was found to be cost-effective compared to the Symptom-only strategy (ICER: ¥185,710/QALY, gaining 0.26 days’ life expectancy). Its cost-effectiveness was significantly influenced by the risks of ovarian and breast cancer among *BRCA1/2* carriers, the prevalence of *BRCA1/2* mutations in the general Chinese population, the prevalence of family history of breast and ovarian cancer among Chinese women, and the prevalence of *BRCA1/2* mutations in the FH-positive population. Integrating these variable distributions, the FH-based strategy showed a 76.96% probability of cost-effectiveness. The Population-based strategy was not cost-effective, whether compared to the Symptom-only strategy (ICER: ¥504,476/QALY, gaining 2.66 days’ life expectancy) or to the FH-based strategy (ICER: ¥539,476/QALY, gaining 2.41 days’ life expectancy). The prevalence of *BRCA1/2* mutations in the general Chinese population was identified as the primary variable affecting its cost-effectiveness. Integrating these variable distributions, the Population-based strategy had a probability of cost-effectiveness of only 0.8%.

**Conclusion:**

Incorporating family-history-based *BRCA1/2* testing into breast and ovarian cancer screening programs is cost-effective in China and warrants promotion.

## 1 Introduction

As China progressively establishes its cancer prevention and screening system, the National Cancer Center of China has consecutively released national cancer statistics for the years 2016 and 2022 (1, 2). These reports reveal that breast cancer is one of the most prevalent cancers among women, with its incidence rate increasing by 42% over the past five years (from 29.5 per 100,000 to 51.17 per 100,000). This significant rise not only imposes a considerable economic burden on healthcare but also highlights trends in cancer development and prevention. These trends are primarily reflected in two aspects: firstly, the aging population phenomenon is leading to a higher incidence of cancer among the older adult; secondly, with the increase in public health awareness and improvements in medical conditions, more people are actively participating in cancer screenings, leading to the early detection of many cancer cases.

Enhancing the detection rate of early-stage breast cancer and its precancerous lesions, followed by timely and effective treatment, is crucial for improving breast cancer prognosis and reducing mortality rates. The Chinese government has initiated several national breast cancer screening programs targeting both the general population and individuals at moderate-to-high risk (3, 4). These programs employ a combination of clinical methods, including visual inspection, physical palpation, and advanced imaging techniques such as breast ultrasonography or mammography. However, none of the existing screening programs have incorporated genetic testing for hereditary mutations, particularly pathogenic variants in the *BRCA1/2* genes, despite their well-established role as major risk factors for breast and ovarian cancer (5–9).

Studies show that approximately 6% of breast cancers can be attributed to hereditary *BRCA1/2* gene mutations (10), and women carrying *BRCA1/2* mutations have a 69–72% risk of developing breast cancer by the age of 80 (8). Women with *BRCA1/2* mutations can opt for prophylactic surgeries (such as mastectomy and salpingo-oophorectomy), chemoprevention, and intensive breast imaging surveillance to reduce their cancer risk (11, 12). Research indicates that chemoprevention can lower the risk of breast cancer by 40–50% (13, 14), mastectomy can reduce the risk by 90–95% (15, 16), and salpingo-oophorectomy can decrease the risk of ovarian cancer by 79–96% (17–19). Therefore, understanding an individual’s genetic mutation status is crucial for the prevention and management of breast cancer. The National Comprehensive Cancer Network (NCCN) has developed various breast cancer risk assessment models based on personal history, family history, age, gender, and germline mutations (20). In recent years, increasing research efforts have been directed towards exploring *BRCA1/2* testing strategies for all breast or ovarian cancer patients and for the general population (21, 22).

However, the cost-effectiveness of *BRCA1/2* testing varies significantly based on the target population, the economic level of the country, and cultural context. Several studies have shown that incorporating *BRCA1/2* testing in population-based breast and ovarian cancer screening is cost-effective in some high-income countries (21, 23). Additionally, including other genes such as *BRCA1*, *BRCA2*, and *PALB2* have also been found to be cost-effective (24–26). In contrast, in middle-income countries like China, the cost-effectiveness of this approach remains uncertain due to several factors. These include a relatively lower GDP per capita, higher costs associated with gene testing and preventive treatments, and varying levels of acceptance of preventive treatments among local women. This study aims to evaluate the feasibility of incorporating *BRCA1/2* testing into breast cancer screening in China by comparing the cost-effectiveness of three strategies: symptom-based screening alone (Symptom-only strategy), population-based *BRCA1/2* testing (population-based strategy), and family-history-based *BRCA1/2* testing (FH-based strategy).

## 2 Materials and methods

### 2.1 Model construction

**Figure 1:**
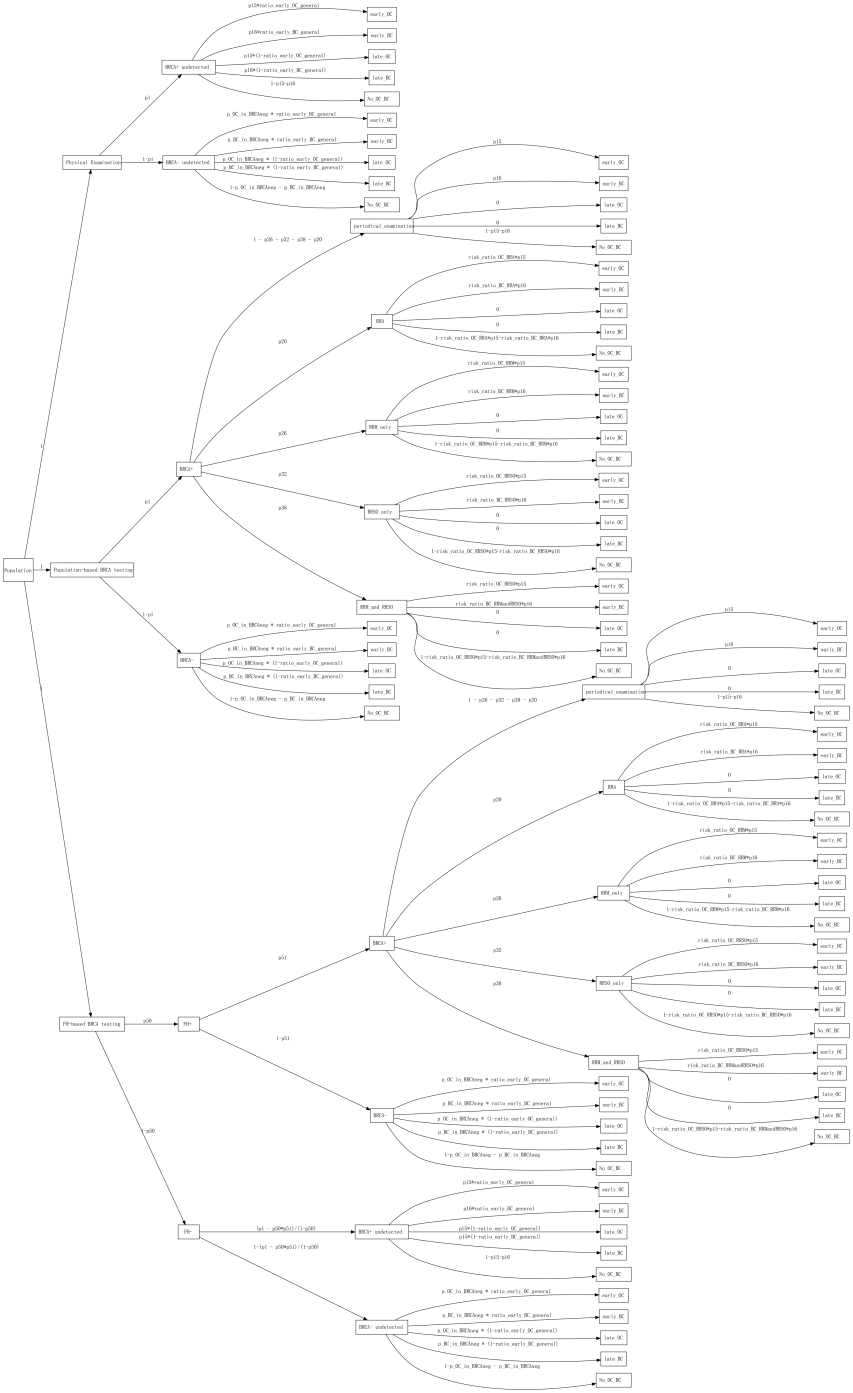
The decision tree model. The boxes contain root, branches, and leaf nodes. The text above the edges represents probabilities or probability formulas.

We constructed a decision tree model comprising three main branches: Symptom-only strategy, Population-based strategy, and FH-based strategy, with five leaf nodes: ovarian cancer detected at early-stage (early_OC), breast cancer detected at early-stage (early_BC), ovarian cancer detected at late-stage (late_OC), breast cancer detected at late-stage (late_BC), and no ovarian or breast cancer detected (Figure 1).

For the Symptom-only strategy, since individuals’ *BRCA1/2* status was unknown, the decision tree branched into BRCA+ undetected and BRCA- undetected based on the frequency of *BRCA1/2* mutations in the population. No further interventions were initiated until events occurred. For the population-based strategy, all individuals underwent *BRCA1/2* gene testing. Those who tested positive for *BRCA1/2* mutations (BRCA+) were further categorized based on intervention measures such as increased surveillance, risk-reducing agents (RRA), and risk-reducing surgeries such as mastectomy (RRM) or salpingo-oophorectomy (RRSO). For those who tested negative for *BRCA1/2* mutations (BRCA−), no further interventions were undertaken. In the FH-based strategy, the decision tree initially branched into FH positive (FH+) and FH negative (FH−) based on family history counseling results. Subsequently, individuals in the FH + branch underwent *BRCA1/2* testing, and intervention measures were implemented based on the test results. Conversely, individuals in the FH− branch did not undergo any intervention until events occurred.

### 2.2 Model parameters

The model encompassed four main categories of parameters: probabilities of events occurring, costs, utilities, and life expectancy.

The model primarily included three types of probabilities: (1) Frequencies of events in the population, including *BRCA1/2* pathogenic mutation frequency in the population (7), FH + population frequency (25), *BRCA1/2* pathogenic mutation frequency among FH + individuals, and proportion of early-stage patients among those who screened positive (27–29). (2) Uptake rates of RRA, RRM, and RRSO in *BRCA1/2* pathogenic mutation carriers (30–32). (3) Risk of developing breast and ovarian cancer by age 70 among carriers of *BRCA1/2* pathogenic mutations and BRCA-negative women (5, 6). These probabilities were derived from articles related to breast and ovarian cancer screening found on PubMed and the China National Knowledge Infrastructure (CNKI).

Our analysis was conducted from a payer’s perspective, incorporating only direct medical care costs obtained from government document (33, 34). All costs were reported in Chinese Yuan (¥). Costs included clinical encounter, ultrasonography testing, and definitive diagnosis of suspected cancer for the Symptom-only strategy. The population-based and FH-based strategies incurred additional costs for genetic testing and family history inquiries. *BRCA1/2* carriers further incurred costs for regular monitoring and preventive treatments.

Quality-adjusted life years (QALYs), which integrate both mortality and health-related quality-of-life effects, were calculated using the formula: QALY = (life-years survived) * utility. Utilities for different health states and surgical interventions were obtained from previous research (35–37). A time horizon of 10 years was chosen based on studies suggesting that this period is sufficient to observe mortality reductions in organized breast cancer screening programs (38). Considering the Chinese Anti-Cancer Association’s recommendation that breast cancer screening should commence at age 40 for individuals at general risk, we extrapolated the 10-year survival rates for breast and ovarian cancer patients diagnosed after age 40 from the SEER database (Supplementary Tables 1, 2; Supplementary Figure 1). We estimated the 10-year survival rates for the general Chinese population using 2019 data from the WHO life table (39).

### 2.3 Model evaluation

To evaluate a decision analysis tree, the expected value of each branch is calculated by multiplying the payoff associated with each transition by the probability of its occurrence, then summing these values. Using this method, we separately computed the Quality-Adjusted Life Years (QALYs) and costs for each of the three strategies. Subsequently, we derived the incremental cost-effectiveness ratio (ICER), which represents the cost per QALY gained. We used three times the Gross Domestic Product (GDP) per capita as the willingness-to-pay (WTP) threshold, i.e., ¥268,200. An ICER greater than this threshold suggests that the strategy lacks cost-effectiveness.

### 2.4 Sensitivity analysis

Sensitivity analysis was employed to explore the uncertainty and robustness of the model results. One-way sensitivity analysis assessed the impact of varying a single parameter on the outcomes. Parameters including probabilities, utilities, and survival rates were varied within their 95% confidence intervals (CIs) where available, or by ±10%. Cost parameters were independently varied by ±30%. Recognizing that model parameters often vary together rather than independently, we also conducted probability sensitivity analysis (PSA). PSA utilized appropriate probability distributions recommended in the literature: beta distributions for probabilities, gamma distributions for costs, and log-normal distributions for utilities and survival rates. The PSA involved running 5,000 iterations of simulation, each time sampling from the distributions of the model parameters. This approach generated 5,000 estimates, allowing us to assess the distribution and uncertainty around the model outcomes comprehensively.

### 2.5 Scenario analysis

Scenario analysis is a useful tool to explore the impact of uncertainties that are not explicitly modeled probabilistically. In our study, we conducted scenario analyses to test key assumptions and their influence on the results. (1) Population prevalence of *BRCA1/2* mutation: We varied this parameter from 0.003965 to 0.00677, aligning it with levels observed in the United States. (2) Risk of developing ovarian cancer: We explored the impact of extending the risk calculation from age 70 to lifelong, setting the risk for *BRCA1/2* carriers at 0.202. (3) Risk of developing breast cancer: Similarly, we analyzed the effect of extending the risk calculation from age 70 to lifelong, with the risk for *BRCA1/2* carriers set at 0.644. (4) Combined risk of ovarian and breast cancer: We examined the scenario where the risks of developing both ovarian and breast cancer were considered simultaneously over a lifetime. (5) Prevalence of FH + in the population: We tested scenarios ranging from 0.0089 to 0.032, using the highest reported rate (40).

### 2.6 Data visualization

We employed three commonly used types of figures to illustrate the cost-effectiveness results: the cost-effectiveness plane, cost-effectiveness acceptability curve (CEAC), and the expected value of perfect information (EVPI) plot. In addition, deterministic sensitivity analyses were depicted using a Tornado diagram. This diagram ranks input parameters in descending order of their impact on model outcomes, illustrating sensitivity to changes in each parameter. All visualizations were created using R packages ggplot2, ggpubr, and survminer.

## 3 Results

### 3.1 Target population and main parameters

This study focused on breast and ovary cancer screening for Chinese women aged 40–60 years, aimed at early diagnosis to improve patient outcomes. The study’s model design and parameter settings were based on this premise. The time horizon was set to 10 years.

The mutation rate of *BRCA1/2* in Chinese women, a crucial factor influencing screening strategies, was set at 0.3965% based on health examination results from 9,331 Han Chinese women (41). A slightly earlier study using next-generation sequencing reported a similar rate of 0.3835% among 1,043 healthy women (40). Of note, an important assumption of this study was the uniform mutation rate of *BRCA1/2* across the entire population for each screening strategy.

The risk of developing breast and ovarian cancer in Chinese women was another pivotal factor in shaping screening strategies. Given our target demographic of women aged 40–60 years over a 10-year period, we considered the probability of developing breast and ovarian cancer by the age of 70, rather than lifetime risk. The cumulative risk of breast cancer by age 70 was derived from the report of Beijing Municipal People’s Government and previous reports for general Chinese women and *BRCA1/2* carriers, respectively (5, 42). Ovarian cancer risks were estimated using a kin-cohort design based on data from 9,903 Chinese breast cancer patients and 3,984 related families (6). These data are also referenced in the “China Expert Consensus on Familial Hereditary Tumors” (43), indicating widespread acceptance among Chinese experts.

A critical parameter was the positivity rate of family history. A study reported a positivity rate of 0.032 for family history of breast and ovarian cancer in healthy Chinese population (40). However, we think this is an outlier. From previous Chinese report (44, 45), we can estimate that family history-positive patients contributed around 28.97% of *BRCA1/2* mutations in breast cancer cases. Assuming a 3.2% positivity rate for family history, family history-positive individuals contributed to 83.44% of *BRCA1/2* mutations in breast cancer cases, approximately 2.88 times higher. Therefore, we adopted previously reported data from the Australian Breast Cancer Family Registry for family history positivity rates, which is 0.0098 (25). Based on this value, family history-positive individuals contributed 25.55% of *BRCA1/2* mutations, aligning reasonably with breast cancer data.

Another important parameter was the 10-year survival rate, based on breast and ovarian cancers diagnosed between 2000 and 2013 in the SEER 8 registries database. This database included 162,253 breast cancer patients and 12,732 ovarian cancer patients aged 40 and above. Patients were categorized into early and late stages based on lymph node metastasis (Supplementary Tables 1, 2). The 10-year survival rates for ovarian cancer were 0.388 (95% CI 0.379–0.398) and 0.176 (95% CI 0.163–0.191) for early and late stages, respectively (Supplementary Figure 1A). For breast cancer, the rates were 0.739 (95% CI 0.736–0.741) and 0.603 (95% CI 0.599–0.607) for early and late stages, respectively (Supplementary Figure 1B). Importantly, these data closely align with an 8-year survival rate observed in a small-scale clinical cohort of breast cancer patients in China (46). Full parameters were specified in Table 1.

**Table 1:** Probabilities, utilities, costs, and parameters used for calculating probabilities in the model.

ID	Value	95%CI	Description	Source
Probability				
p1	0.003965	0.002690–0.005240	*BRCA1/2* mutation prevalence in general women	(41)
p15	0.130385	0.069231–0.166615	Risk of developing ovary cancer in *BRCA1/2* carriers	(6, 7)
p16	0.374000	0.333–0.434	Risk of developing breast cancer in *BRCA1/2* carriers	(5)
p_BC_general	0.036000		Risk of developing ovary cancer in general women	(5, 43)
p_OC_in_BRCAneg	0.004000	0.003–0.007	Risk of developing ovary cancer in *BRCA1/2* non-carrier	(6)
p_BC_in_BRCAneg	0.034654		risk of developing breast cancer in *BRCA1/2* non-carrier	(5)
ratio_early_OC_general	0.300000		Ratio of early-stage cancer in ovary cancer screened	(27)
ratio_early_BC_general	0.621000		Ratio of early-stage cancer in breast cancer screened	(28, 29)
p20	0.163000	0.136–0.19	Uptake rate of chemoprevention in *BRCA1/2* carriers	(14)
p26	0.211500	0.0815–0.3015	Uptake rate of RRM-only in *BRCA1/2* carriers	(30)
p32	0.291500	0.0415–0.4915	Uptake rate of RRSO-only in *BRCA1/2* carriers	(50)
p38	0.258500		Uptake rate of both RRSO and RRM in *BRCA1/2* carriers	(30, 50)
risk_ratio_OC_RRA	0.530000	0.41–0.67	Risk ratio for ovary cancer from RRA in *BRCA1/2* carriers	(13)
risk_ratio_BC_RRA	0.640000	0.40–1.03	Risk ratio for breast cancer from RRA in *BRCA1/2* carriers	(14)
risk_ratio_OC_RRM	1.000000		Risk ratio for ovary cancer from RRM in *BRCA1/2* carriers	
risk_ratio_BC_RRM	0.114000	0.041–0.317	Risk ratio for breast cancer from RRM in *BRCA1/2* carriers	(31)
risk_ratio_OC_RRSO	0.210000	0.12–0.39	Risk ratio for ovary cancer from RRSO in *BRCA1/2* carriers	(32)
risk_ratio_BC_RRSO	0.490000	0.37–0.65	Risk ratio for breast cancer from RRSO in *BRCA1/2* carriers	(32)
risk_ratio_BC_RRMandRRSO	0.050000	0.01–0.22	Risk ratio for breast cancer from RRM and RRSO in *BRCA1/2* carriers	(16)
p50	0.009800	0.0047–0.0179	Prevalence of family history of OC/BC in general population	(25)
p51	0.100000		*BRCA1/2* mutation prevalence in population with family history of OC/BC	
Utility				
early_OC	0.81		Utility for early-stage ovary cancer	(35)
early_BC	0.71		Utility for early-stage breast cancer	(36)
late_OC	0.55		Utility for advance-stage ovary cancer	(35)
late_BC	0.65		Utility for advance-stage breast cancer	(36)
No_OC_BC	1		Utility for health women	
RRM (year 1)	0.88		First year utility of RRM	(36, 37)
RRSO (year 1)	0.95		First year utility of RRSO	(36, 37)
10-years survival rate				
early_OC	0.388	0.379–0.398	10-years survival rate for early-stage ovary cancer	
early_BC	0.739	0.736–0.741	10-years survival rate for early-stage breast cancer	
late_OC	0.176	0.163–0.191	10-years survival rate for advance-stage ovary cancer	
late_BC	0.603	0.599–0.607	10-years survival rate for advance-stage breast cancer	
No_OC_BC	0.977658738		10-years survival rate for health women	(39)
Cost, ¥				
cost_general_examination	400		Cost of clinical encounter and ultrasonography	(34)
cost_periodical_examination	1,500		cost of mammography for 3 consecutive years	(34)
cost_FH_consel	80		Cost of family history counseling	(34)
cost_brca_test	3,600		Cost of BRCA testing	(33)
cost_rra	536		Cost of RRA	(26)
cost_rrm	5,414		Cost of RRM	(26)
cost_rrso	26,881		Cost of RRSO	(26)
cost_hrt	4,200		Cost of hormone replacement therapy (HRT)	(52)
cost_diagnosis_cancer	1,600		Cost of definitive diagnosis of cancer	(34)

### 3.2 Base-case analysis

In the study, the three strategies (Symptom-only strategy, Population-based strategy, and FH-based strategy) incurred total costs of ¥465, ¥4,142, and ¥599, respectively. The corresponding QALYs were 9.5587, 9.5660, and 9.5594 years (Table 2). Compared to the Symptom-only strategy, the Population-based strategy extended QALYs by 0.0073 years, equivalent to 2.66 days, at an additional cost of ¥3,677. This resulted in an ICER of 504,476, which was 1.88 times higher than the WTP threshold of ¥268,200. Compared to the FH-based strategy, the Population-based strategy gained an additional 0.0066 years (2.41 days) of QALYs at an extra cost of ¥3,543, resulting in an ICER of 539,476, which exceeded twice the WTP threshold.

**Table 2:** Base-case analysis results.

Strategy	Cost, ¥	QALY	ICER, ¥/QALY
Symptom-only strategy	465	9.5587	Control
Population-based BRCA testing strategy	4,142	9.5660	504,476
FH-based BRCA testing strategy	599	9.5594	185,710

Importantly, when comparing the FH-based strategy with the Symptom-only strategy, an ICER of 185,710 was derived. The FH-based strategy gained 0.0007 years (0.26 days) of QALYs at an additional cost of ¥134, indicating that the FH-based strategy was the most cost-effective option.

### 3.3 Sensitivity analysis

#### 3.3.1 One-way sensitivity analyses

**Figure 2:**
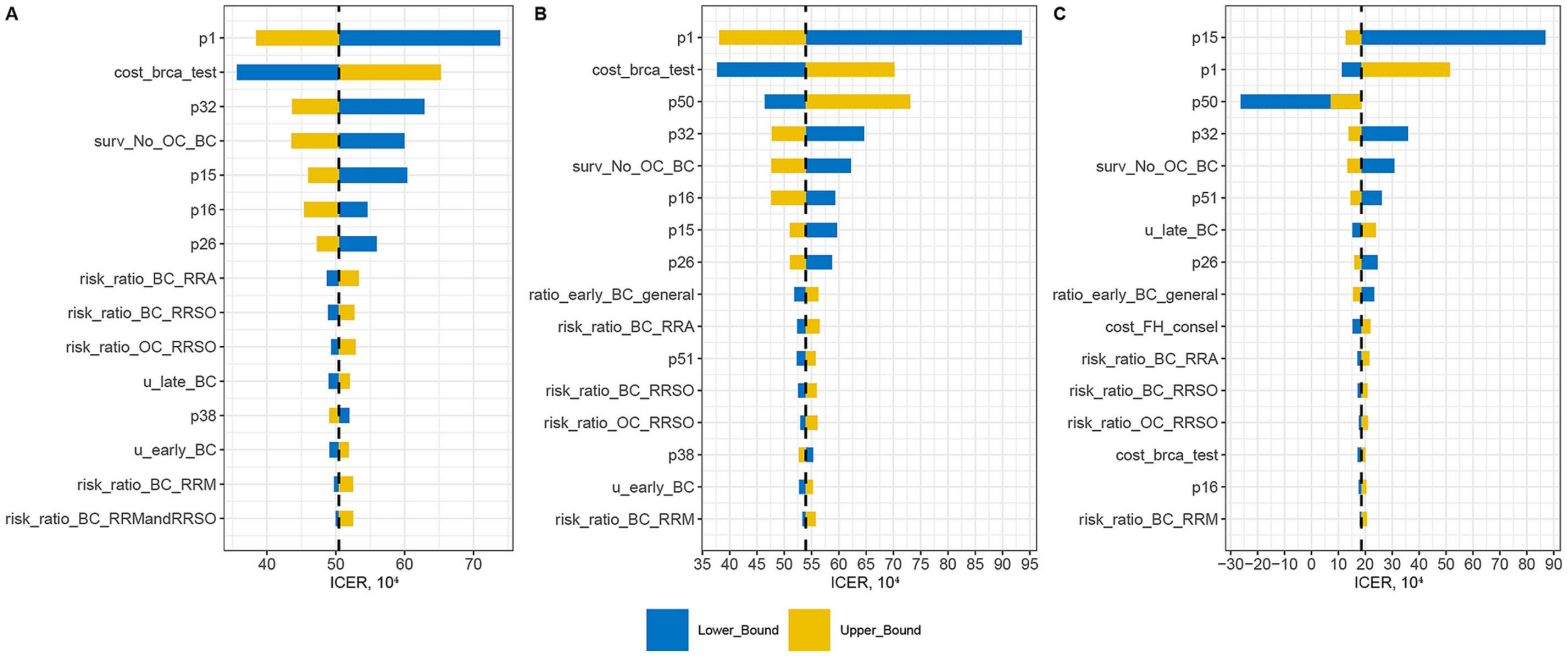
One-way sensitivity analysis assessing impact of variables on three sets of ICERs: Population-based strategy versus Symptom-only **(A)**, Population-based strategy versus FH-based strategy **(B)**, and FH-based strategy versus Symptom-only **(C)**.

A total of 42 variables were analyzed to assess their impact on three sets of ICERs: Population-based strategy versus Symptom-only (Figure 2A), Population-based strategy versus FH-based strategy (Figure 2B), and FH-based strategy versus Symptom-only (Figure 2C).

The variable with the greatest impact on the ICER of the Population-based strategy was the prevalence of *BRCA1/2* mutation in the general Chinese women (p1). A higher p1 value corresponded to greater cost-effectiveness of the Population-based strategy. Other significant probability factors included breast cancer risk for *BRCA1/2* carriers (p16), ovarian cancer risk for *BRCA1/2* carriers (p15), uptake rate of RRM (p26), and uptake rate of both RRM and RRSO in *BRCA1/2* carriers (p32). Higher values of these variables also enhanced the cost-effectiveness of the Population-based strategy. Among cost variables, the cost of *BRCA1/2* testing was the second most influential factor on the ICER of the Population-based strategy. However, even with a 30% reduction in testing costs, the Population-based strategy remained cost-ineffective. Further analysis indicated that the strategy became cost-effective only when testing costs decreased by 48% (i.e., ¥1,878).

For the ICER of the FH-based strategy, the variable with the greatest impact was the ovarian cancer risk for *BRCA1/2* carriers (p15). An increase in p15 significantly raised the ICER, while a decrease had a less pronounced effect. The prevalence of *BRCA1/2* mutation in general Chinese women (p1), the prevalence of breast/ovary cancer family history in general Chinese women (p50), and the *BRCA1/2* mutation prevalence in FH + population (p51) were interrelated and affected the ICER of the FH-based strategy. Generally, the ICER decreased with an increase in the FH + rate. However, when the FH + rate was too low (<0.0065), a high *BRCA1/2* + rate in the FH− population led to lower QALYs compared to the Symptom-only strategy, resulting in a negative ICER (Supplementary Figure 2). Besides, the cost of *BRCA1/2* testing had a minimal impact on the ICER of the FH-based strategy.

#### 3.3.2 Probabilistic sensitivity analysis

**Figure 3:**
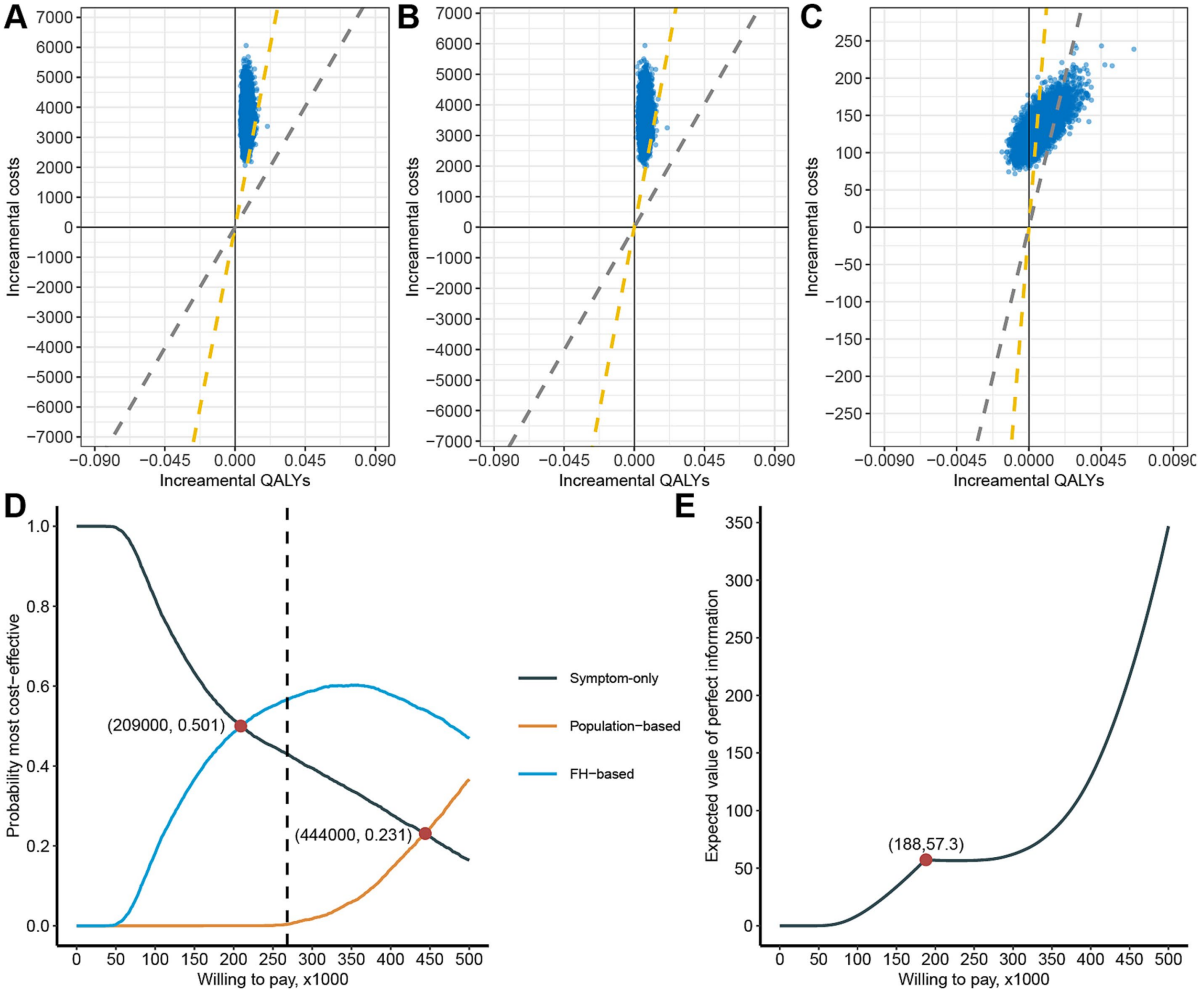
Probability sensitivity analysis of cost-effectiveness of the three strategies. Cost-effective planes of population-based strategy versus Symptom-only **(A)**, Population-based strategy versus FH-based strategy **(B)**, and FH-based strategy versus Symptom-only **(C)**, The yellow dashed diagonal line represents WTP cutoff. Cost-effectiveness acceptability curve of the three strategies **(D)**. The expected value of perfect information plot **(E)**.

We conducted a Monte Carlo simulation 5,000 times using variables generated from corresponding distributions. We evaluated three strategies: Population-based strategy versus Symptom-only strategy (Figure 3A), Population-based strategy versus FH-based strategy (Figure 3B), and FH-based strategy versus Symptom-only strategy (Figure 3C). The probability of being cost-effective for these strategies were 0.74, 0.80, and 76.96%, respectively.

From a net monetary benefit perspective, the FH-based strategy becomes economically favorable compared to the Symptom-only strategy when WTP exceeds ¥209,000, emerging as the most cost-effective strategy (Figure 3D). Similarly, the Population-based strategy begins to surpass the Symptom-only strategy economically when WTP exceeds ¥444,000, although it remains less cost-effective than the FH-based strategy. Notably, at the current WTP threshold, the probabilities of being most cost-effective are 43.00% for the Symptom-only strategy, 0.34% for the Population-based strategy, and 56.6% for the FH-based strategy.

Since cost-effectiveness acceptability curves do not account for the magnitude of cost and QALY gains, we conducted an expected value of perfect information (EVPI) analysis. The EVPI increases with higher WTP thresholds, reaching a local maximum of 57.3 at WTP equal to ¥188,000 (Figure 3E). This suggests that consumers have a higher willingness to pay to eliminate uncertainty at this specific WTP threshold.

### 3.4 Scenario result

Given that the cost of *BRCA1/2* testing is a controllable variable, we examined two critical scenarios (Table 3). When the cost of *BRCA1/2* testing decreases to ¥1,878 (a 48% reduction), the Population-based strategy becomes cost-effective compared to the Symptom-only strategy. Further reduction in testing costs to ¥1,808 (a 50% reduction) makes the Population-based strategy cost-effective compared to the FH-based strategy. Conversely, the cost of *BRCA1/2* testing has a minimal impact on the ICER of the FH-based strategy versus Symptom-only strategy, only becoming cost-ineffective when costs exceed ¥9,670.

**Table 3:** Scenario analysis.

Scenario	Symptom-based strategy		Population-based strategy		FH-based strategy		ICER		
	Cost	QALY	Cost	QALY	Cost	QALY	Population-based versus symptom-only	Population-based versus FH-based	FH-based versus symptom-only
Adjust cost of *BRCA1/2* testing									
Cutoff at ¥1,878	465	9.558696	2,420	9.565985	582	9.559417	268,200	279,827	162,305
Cutoff at ¥1,808	465	9.558696	2,350	9.565985	581	9.559417	258,575	269,250	161,352
Increase risk of cancer to lifetime									
p15 to 0.202	465	9.556379	4,142	9.565071	599	9.557765	423,062	484,937	96,729
p16 to 0.644	467	9.553307	4,143	9.564246	600	9.553733	336,059	337,004	312,719
p15 and p16	467	9.550991	4,143	9.563332	598	9.552081	297,857	314,870	122,241
Use the FH rate reported in China									
FH + rate to 0.32%	465	9.558696	4,142	9.565985	722	9.564301	504,476	2,031,108	45,921

Considering our estimates of the probability of *BRCA1/2* carriers developing ovarian cancer (p15) and breast cancer (p16) until age 70, which are significantly lower than lifetime rates reported in other studies, we explored three scenarios (Table 3): increasing p15 to 0.202, increasing p16 to 0.644, and increasing both p15 to 0.202 and p16 to 0.644. In all three scenarios, the ICER of the Population-based strategy decreased significantly, with the third scenario showing an ICER of ¥297,857, approaching cost-effectiveness.

Finally, we examined an extreme scenario where the prevalence of positive family history increased to 0.32% (Table 3). This did not affect the ICER of the Population-based strategy compared to the Symptom-only strategy. However, it significantly improved the cost-effectiveness of the FH-based strategy (ICER of Population-based strategy against FH-based strategy increased to ¥2,031,108, and ICER of FH-based strategy against Population-based strategy decreased to ¥45,921).

## 4 Discussion

In China, the women’s cancer screening program has been operational for several years, primarily utilizing basic clinical visual inspection, palpation, and breast color Doppler ultrasound. Given the proven impact of *BRCA1/2* germline mutations on the incidence of breast and ovarian cancer, integrating *BRCA1/2* mutation testing into screening programs has become imperative. However, whether incorporating it into these programs is cost-effective, either for the entire population or through enrichment based on family history of breast and ovary cancer, remains poorly researched. This study explored the cost-effectiveness of three strategies: Symptom-only strategy, Population-based strategy, and FH-based strategy, for breast and ovarian cancer screening in women aged 40–60. It was found that under a threshold of 3 times GDP per capita, the FH-based strategy demonstrated economic viability, with a probability of 73.42%. Its cost-effectiveness was influenced significantly by the proportion of *BRCA1/2* mutation carriers, the risk of developing ovary cancer in *BRCA1/2* carriers, and the proportion of FH-positive individuals, while factors such as the cost of *BRCA1/2* testing had a lesser impact. When considering lifetime cancer risks, the FH-based strategy showed even greater economic efficiency. Conversely, the Population-based strategy did not demonstrate cost-effectiveness.

The integration of *BRCA1/2* mutation testing into female breast and ovarian cancer screening has been extensively researched and deemed cost-effective, predominantly based on data from developed countries like the US and UK (24, 25, 47, 48). Studies specific to middle-income countries, such as those using data from Mexico and Brazil, differ significantly from Chinese data (23, 47). Sensitivity analyses highlighted the *BRCA1/2* mutation frequency as a critical variable. In China, the *BRCA1/2* mutation frequency is moderate globally, lower than in the Ashkenazi Jewish population (2.17%) and higher than in Japanese (0.26%), Malaysians (0.18%), and the Mexican population (0.38%) (41, 49). Moreover, the types of *BRCA1/2* mutations also differ substantially from foreign data (41, 44), likely contributing to discrepancies in breast and ovarian cancer risk for *BRCA1/2* carriers in China. The breast and ovarian cancer incidence rates among Chinese women up to age 70 are 37.40 and 13.04%, respectively, which are more applicable to our target population (women aged 40–60) and notably lower than lifetime risk used in most literature (24–26). When considering lifetime risks, the FH-based strategy became more economically viable, while the Population-based strategy did not.

Decision trees and Markov chains are commonly used models in health economics research. Prior studies using Markov chain models have typically focused on patient outcomes rather than cancer diagnosis endpoints (23, 26, 47), diverging from our study’s focus on prevention through large-scale screening to identify *BRCA1/2* carriers and reduce cancer incidence probability through intensive follow-up and intervention. Defining cancer diagnosis as an endpoint (leaf nodes) better reflects this objective. Furthermore, our study refined the endpoint by distinguishing cancer diagnoses into early and late stages to further reflect the value of early detection.

Our study considered data accuracy and model applicability comprehensively. Nevertheless, some crucial factors influencing conclusions are beyond our control. Foremost is the accuracy of the proportion of FH-positive individuals. We utilized data from Australia, which is logically more reasonable but not directly applicable to China, hence hindering precise results. Despite reports on the proportion of FH-positive patients among breast cancer patients, comprehensive data on FH-positive individuals across the entire population are lacking. Moreover, the probability of *BRCA1/2* carriers undergoing preventive treatment was based on previous literature (14, 30, 50), lacking precise Chinese data. Nonetheless, only when the uptake rate of RRSO in *BRCA1/2* carriers drop to 0.1243 (a decrease of 57.36%), does the FH-based strategy no longer exhibit cost-effectiveness. Thus, it is highly unlikely to overturn our conclusion. Additionally, we did not account for the risk of synchronous breast and ovarian cancers in *BRCA1/2* carriers. Although the incidence of double primaries (0.27%) is relatively low, and the probability of synchronous diagnoses (within one year) accounts for 32.75% of cases (derived from SEER). This may have led to a slight overestimation of breast and ovarian cancer risks. Furthermore, we treated carriers of *BRCA1/2* variants of uncertain significance (VUS) as having the same risk as *BRCA1/2* wild-type individuals, consistent with current guidelines that classify VUS as uninformative for cancer risk stratification and management (20). However, the inherent uncertainty surrounding VUS reflects limitations in current knowledge and data (51). We expect that future studies with more comprehensive VUS characterization will enable a more accurate assessment of their clinical and health-economic implications. Finally, given the complexity of therapeutic interventions, our current study is intentionally focused exclusively on the domain of cancer prevention. We have not included economic evaluations of various post-diagnosis treatment strategies currently. Consequently, our analysis does not yet enable comprehensive health economic assessments covering the full lifecycle of prevention and treatment for *BRCA1/2* mutation carriers.

Overall, in breast and ovarian cancer screening programs for women in China, incorporating *BRCA1/2* genetic testing for individuals with a family history, along with appropriate preventive measures for those who test positive, increases the population’s QALY by 0.26 days. The corresponding ICER is ¥185,710, which is well below three times the per capita GDP, indicating cost-effectiveness and making it worthy of promotion in China.

## Data Availability

The original contributions presented in the study are included in the article/Supplementary material, further inquiries can be directed to the corresponding authors.

## Supplementary material

SUPPLEMENTARY FIGURE 1 Kaplan-Meier survival analysis of ovary cancer **(A)** and breast cancer **(B)** patients aged 40 and above in SEER database. The two curves represent the negative and positive lymph node metastasis groups.

SUPPLEMENTARY FIGURE 2 ICER of FH-based strategy versus Symptom-only varies with changes in the family history positivity rates of breast and ovarian cancer. As x approaches 0.0065, y approaches infinity.

## References

[1] Zheng RS Chen R Han BF Wang SM Li L Sun KX. Cancer incidence and mortality in China, 2022. Zhonghua Zhong Liu Za Zhi. (2024) 46:221–31. doi: 10.3760/cma.j.cn112152-20240119-00035, PMID: 38468501

[2] Zheng RS Zhang SW Sun KX Chen R Wang SM Li L. Cancer statistics in China, 2016. Zhonghua zhong liu za zhi [Chinese journal of oncology]. (2023) 45:212–20. doi: 10.3760/cma.j.cn112152-20220922-00647, PMID: 36944542

[3] He J Chen WQ Li N Shen HB Li J Wang Y. China guideline for the screening and early detection of female breast cancer. Zhonghua Zhong Liu Za Zhi. (2021) 43:357–82. doi: 10.3760/cma.j.cn112152-20210119-00061 33902200

[4] The Society of Breast Cancer China Anti-Cancer Association BOGotOBotCMA. Guidelines for breast cancer diagnosis and treatment by China anti-cancer association (2024 edition). China Oncol. (2024) 33:1092–187. doi: 10.19401/j.cnki.1007-3639.2023.12.004

[5] Yao L Sun J Zhang J He Y Ouyang T Li J. Breast cancer risk in Chinese women with BRCA1 or BRCA2 mutations. Breast Cancer Res Treat. (2016) 156:441–5. doi: 10.1007/s10549-016-3766-3, PMID: 27033093

[6] Yao L Sun J Hu L Chen J Zhang J Xu Y. Ovarian cancer risk of Chinese women with BRCA1/2 germline pathogenic variants. J Hum Genet. (2022) 67:639–42. doi: 10.1038/s10038-022-01065-6, PMID: 35864222

[7] Shi T Wang P Xie C Yin S Shi D Wei C. BRCA1 and BRCA2 mutations in ovarian cancer patients from China: ethnic-related mutations in BRCA1 associated with an increased risk of ovarian cancer. Int J Cancer. (2017) 140:2051–9. doi: 10.1002/ijc.30633, PMID: 28176296

[8] Kuchenbaecker KB Hopper JL Barnes DR Phillips KA Mooij TM Roos-Blom MJ. Risks of breast, ovarian, and contralateral breast cancer for BRCA1 and BRCA2 mutation carriers. JAMA. (2017) 317:2402–16. doi: 10.1001/jama.2017.7112, PMID: 28632866

[9] Bolze A Cirulli ET Hajek C Schnell Blitstein JM Grzymski JJ. The potential of genetics in identifying women at lower risk of breast cancer. JAMA Oncol. (2024) 10:236. doi: 10.1001/jamaoncol.2023.5468, PMID: 38153744 PMC10870185

[10] Buys SS Sandbach JF Gammon A Patel G Kidd J Brown KL. A study of over 35,000 women with breast cancer tested with a 25-gene panel of hereditary cancer genes. Cancer. (2017) 123:1721–30. doi: 10.1002/cncr.30498, PMID: 28085182

[11] Domchek SM Friebel TM Singer CF Evans DG Lynch HT Isaacs C. Association of risk-reducing surgery in BRCA1 or BRCA2 mutation carriers with cancer risk and mortality. JAMA. (2010) 304:967–75. doi: 10.1001/jama.2010.1237, PMID: 20810374 PMC2948529

[12] De Felice F Marchetti C Musella A Palaia I Perniola G Musio D. Bilateral risk-reduction mastectomy in BRCA1 and BRCA2 mutation carriers: a meta-analysis. Ann Surg Oncol. (2015) 22:2876–80. doi: 10.1245/s10434-015-4532-1, PMID: 25808098

[13] Park J Huang D Chang YJ Lim MC Myung SK. Oral contraceptives and risk of breast cancer and ovarian cancer in women with a BRCA1 or BRCA2 mutation: a meta-analysis of observational studies. Carcinogenesis. (2022) 43:231–42. doi: 10.1093/carcin/bgab107, PMID: 34958358

[14] Kotsopoulos J Gronwald J Huzarski T Aeilts A Randall Armel S Karlan B. Tamoxifen and the risk of breast cancer in women with a BRCA1 or BRCA2 mutation. Breast Cancer Res Treat. (2023) 201:257–64. doi: 10.1007/s10549-023-06991-3, PMID: 37432545

[15] Nelson HD Pappas M Zakher B Mitchell JP Okinaka-Hu L Fu R. Risk assessment, genetic counseling, and genetic testing for BRCA-related cancer in women: a systematic review to update the U.S. preventive services task force recommendation. Ann Intern Med. (2014) 160:255–66. doi: 10.7326/M13-1684 24366442

[16] Rebbeck TR Friebel T Lynch HT Neuhausen SL van 't Veer L Garber JE. Bilateral prophylactic mastectomy reduces breast cancer risk in BRCA1 and BRCA2 mutation carriers: the PROSE study group. J Clin Oncol. (2004) 22:1055–62. doi: 10.1200/JCO.2004.04.188 14981104

[17] Finch A Beiner M Lubinski J Lynch HT Moller P Rosen B. Salpingo-oophorectomy and the risk of ovarian, fallopian tube, and peritoneal cancers in women with a BRCA1 or BRCA2 mutation. JAMA. (2006) 296:185–92. doi: 10.1001/jama.296.2.185 16835424

[18] Gould Rothberg BE Bracken MB Rimm DL. Tissue biomarkers for prognosis in cutaneous melanoma: a systematic review and meta-analysis. J Natl Cancer Inst. (2009) 101:452–74. doi: 10.1093/jnci/djp038 19318635 PMC2720709

[19] Kauff ND Domchek SM Friebel TM Robson ME Lee J Garber JE. Risk-reducing salpingo-oophorectomy for the prevention of BRCA1- and BRCA2-associated breast and gynecologic cancer: a multicenter, prospective study. J Clin Oncol. (2008) 26:1331–7. doi: 10.1200/JCO.2007.13.9626, PMID: 18268356 PMC3306809

[20] The National Comprehensive Cancer Network (NCCN). Genetic/familial high-risk assessment: breast, ovarian, and pancreatic and prostate (2024).

[21] D'Andrea E Marzuillo C De Vito C Di Marco M Pitini E Vacchio MR. Which BRCA genetic testing programs are ready for implementation in health care? A systematic review of economic evaluations. Genet Med. (2016) 18:1171–80. doi: 10.1038/gim.2016.29, PMID: 27906166 PMC5159446

[22] Koldehoff A Danner M Civello D Rhiem K Stock S Müller D. Cost-effectiveness of targeted genetic testing for breast and ovarian Cancer: a systematic review. Value Health. (2021) 24:303–12. doi: 10.1016/j.jval.2020.09.016, PMID: 33518037

[23] Manchanda R Sun L Patel S Evans O Wilschut J De Freitas Lopes AC. Economic evaluation of population-based BRCA1/BRCA2 mutation testing across multiple countries and health systems. Cancers. (2020) 12:1929. doi: 10.3390/cancers12071929, PMID: 32708835 PMC7409094

[24] Guo F Adekanmbi V Hsu CD Berenson AB Kuo Y-F Shih Y-CT. Cost-effectiveness of population-based multigene testing for breast and ovarian Cancer prevention. JAMA Netw Open. (2024) 7:e2356078. doi: 10.1001/jamanetworkopen.2023.56078, PMID: 38353949 PMC10867683

[25] Manchanda R Patel S Gordeev VS Antoniou AC Smith S Lee A. Cost-effectiveness of population-based BRCA1, BRCA2, RAD51C, RAD51D, BRIP1, PALB2 mutation testing in unselected general population women. JNCI J Natl Cancer Inst. (2018) 110:714–25. doi: 10.1093/jnci/djx265, PMID: 29361001

[26] Sun L Cui B Wei X Sadique Z Yang L Manchanda R. Cost-effectiveness of genetic testing for all women diagnosed with breast Cancer in China. Cancers (Basel). (2022) 14:1839. doi: 10.3390/cancers14071839, PMID: 35406611 PMC8997428

[27] Wang Y Wang Z Zhang Z Wang H Peng J Hong L. Burden of ovarian cancer in China from 1990 to 2030: a systematic analysis and comparison with the global level. Front Public Health. (2023) 11:1136596. doi: 10.3389/fpubh.2023.1136596 36860393 PMC9969192

[28] Li M Wang H Qu N Piao H Zhu B. Breast cancer screening and early diagnosis in China: a systematic review and meta-analysis on 10.72 million women. BMC Womens Health. (2024) 24:97. doi: 10.1186/s12905-024-02924-4, PMID: 38321439 PMC10848517

[29] Zheng R Wang S Zhang S Zeng H Chen R Sun K. Global, regional, and national lifetime probabilities of developing cancer in 2020. Sci Bull. (2023) 68:2620–8. doi: 10.1016/j.scib.2023.09.041, PMID: 37821267 PMC10640926

[30] Evans DG Lalloo F Ashcroft L Shenton A Clancy T Baildam AD. Uptake of risk-reducing surgery in unaffected women at high risk of breast and ovarian cancer is risk, age, and time dependent. Cancer Epidemiol Biomarkers Prev. (2009) 18:2318–24. doi: 10.1158/1055-9965.EPI-09-0171, PMID: 19661091

[31] Li X You R Wang X Liu C Xu Z Zhou J. Effectiveness of prophylactic surgeries inBRCA1orBRCA2Mutation carriers: a Meta-analysis and systematic review. Clin Cancer Res. (2016) 22:3971–81. doi: 10.1158/1078-0432.CCR-15-1465, PMID: 26979395

[32] Rebbeck TR Kauff ND Domchek SM. Meta-analysis of risk reduction estimates associated with risk-reducing salpingo-oophorectomy in BRCA1 or BRCA2 mutation carriers. J Natl Cancer Inst. (2009) 101:80–7. doi: 10.1093/jnci/djn442, PMID: 19141781 PMC2639318

[33] 上海市医疗保障局. 关于剬布本市部分新增医疗服务项目价格和可另收费一次性使用医疗器械目录的通知. Available online at: https://ybj.sh.gov.cn/gsgg/20201109/296b2dbc699f495e9927ded43a8b6786.html.

[34] 上海市医疗保障局. (2021). 关于进一步规范磁共振扫描等医疗服务价格项目的通知沪医保价采发 33 号. Available online at: https://ybj.sh.gov.cn/qtwj/20211104/b0e74c826d424e339e1094a7ed0144c7.html.

[35] Havrilesky LJ Broadwater G Davis DM Nolte KC Barnett JC Myers ER. Determination of quality of life-related utilities for health states relevant to ovarian cancer diagnosis and treatment. Gynecol Oncol. (2009) 113:216–20. doi: 10.1016/j.ygyno.2008.12.026, PMID: 19217148 PMC2713675

[36] Sun L Brentnall A Patel S Buist DSM Bowles EJA Evans DGR. A cost-effectiveness analysis of multigene testing for all patients with breast Cancer. JAMA Oncol. (2019) 5:1718–30. doi: 10.1001/jamaoncol.2019.3323, PMID: 31580391 PMC6777250

[37] Williams-Frame A Carpenter JS. Costs of hormonal and nonhormonal prescription medications for hot flashes. Women's Health (Lond Engl). (2009) 5:497–502. doi: 10.2217/WHE.09.49, PMID: 19702449 PMC3637932

[38] Wang J Greuter MJW Zheng S van Veldhuizen DWA Vermeulen KM Wang Y. Assessment of the benefits and cost-effectiveness of population-based breast Cancer screening in urban China: a model-based analysis. Int J Health Policy Manag. (2022) 11:1658–67. doi: 10.34172/ijhpm.2021.62, PMID: 34273933 PMC9808213

[39] WHO Life table, China. (2019). Availabe online at: https://wwwwhoint/data/gho/data/indicators/indicator-details/GHO/gho-ghe-life-tables-by-country

[40] Lang GT Shi JX Hu X Zhang CH Shan L Song CG. The spectrum of BRCA mutations and characteristics of BRCA-associated breast cancers in China: screening of 2,991 patients and 1,043 controls by next-generation sequencing. Int J Cancer. (2017) 141:129–42. doi: 10.1002/ijc.30692, PMID: 28294317

[41] Dong H Chandratre K Qin Y Zhang J Tian X Rong C. Prevalence ofBRCA1/BRCA2pathogenic variation in Chinese Han population. J Med Genet. (2021) 58:565–9. doi: 10.1136/jmedgenet-2020-106970, PMID: 32467295

[42] 北京市政府. “2013年北京市卫生与人群健康状况报告” 发布[J]. 健康. Beijing municipal health and population health report (2014) 80–1.

[43] 中国抗癌协会家族遗传性肿瘤专业委员会. (2021). 中 国家族遗传性肿瘤临床诊疗专家共识(2021年版). Zhongguo Zhong Liu Lin Chuang. 48(23):7.

[44] Gao X Nan X Liu Y Liu R Zang W Shan G. Comprehensive profiling of BRCA1 and BRCA2 variants in breast and ovarian cancer in Chinese patients. Hum Mutat. (2019) 41:696–708. doi: 10.1002/humu.23965 31825140

[45] Liu L Hao X Song Z Zhi X Zhang S Zhang J. Correlation between family history and characteristics of breast cancer. Sci Rep. (2021) 11:6360. doi: 10.1038/s41598-021-85899-8, PMID: 33737705 PMC7973811

[46] Peng Z Wei J Lu X Zheng H Zhong X Gao W. Treatment and survival patterns of Chinese patients diagnosed with breast cancer between 2005 and 2009 in Southwest China. Medicine. (2016) 95:e3865. doi: 10.1097/MD.0000000000003865, PMID: 27336872 PMC4998310

[47] Lourenção M Simões Correa Galendi J Galvão HCR Antoniazzi AP Grasel RS Carvalho AL. Cost-effectiveness of BRCA 1/2 genetic test and preventive strategies: using real-world data from an upper-middle income country. Front Oncol. (2022) 12:951310. doi: 10.3389/fonc.2022.951310 35898894 PMC9309566

[48] Wei X Sun L Slade E Fierheller CT Oxley S Kalra A. Cost-effectiveness of gene-specific prevention strategies for ovarian and breast Cancer. JAMA Netw Open. (2024) 7:e2355324. doi: 10.1001/jamanetworkopen.2023.55324, PMID: 38334999 PMC10858404

[49] Lei H Zhang M Zhang L Hemminki K Wang X-j Chen T. Overview on population screening for carriers with germline BRCA mutation in China. Front Oncol. (2022) 12:1002360. doi: 10.3389/fonc.2022.1002360 36439508 PMC9682265

[50] Manchanda R Burnell M Abdelraheim A Johnson M Sharma A Benjamin E. Factors influencing uptake and timing of risk reducing salpingo-oophorectomy in women at risk of familial ovarian cancer: a competing risk time to event analysis. BJOG. (2012) 119:527–36. doi: 10.1111/j.1471-0528.2011.03257.x, PMID: 22260402

[51] 中国抗癌协会肿瘤标志专业委员会 上. 基于中国人群的BRCA胚系突变筛查专家共识(2024年版). Chna Oncology (2024);34(2):220–238.

[52] 中国抗癌协会肿瘤内分泌专业委员会, 周, 琦, 邹, 冬玲, 张, 师前. 妇科恶性肿瘤激素补充治疗中国专家共识(2024年版). Zhongguo Shi Yong Fu Ke Yu Chan Ke Za Zhi (2024);40(3):305–311.

