# Supplementary figures and images for: Cost-effectiveness analysis of population-based BRCA1/2 testing, family-history-based BRCA1/2 testing, and symptom-based screening for breast and ovarian cancer in China
Source: Front Public Health. 2025 Jun 11;13:1479966. doi: 10.3389/fpubh.2025.1479966 (PMC12187657; doi:10.3389/fpubh.2025.1479966)

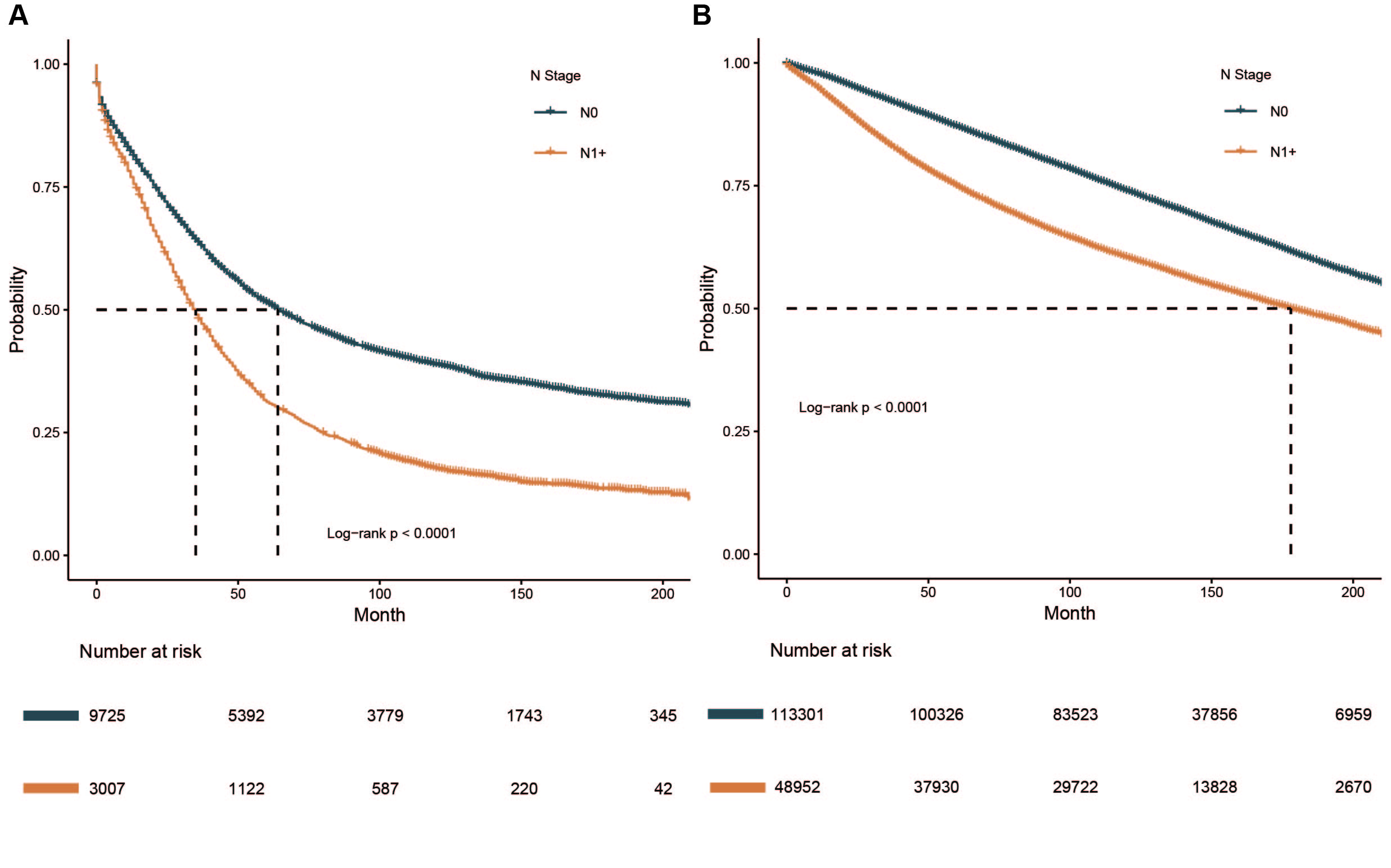

Supplement: SUPPLEMENTARY FIGURE 1 — Kaplan-Meier survival analysis of ovary cancer (A) and breast cancer (B) patients aged 40 and above in SEER database. The two curves represent the negative and positive lymph node metastasis groups. [file Image_1.jpeg]

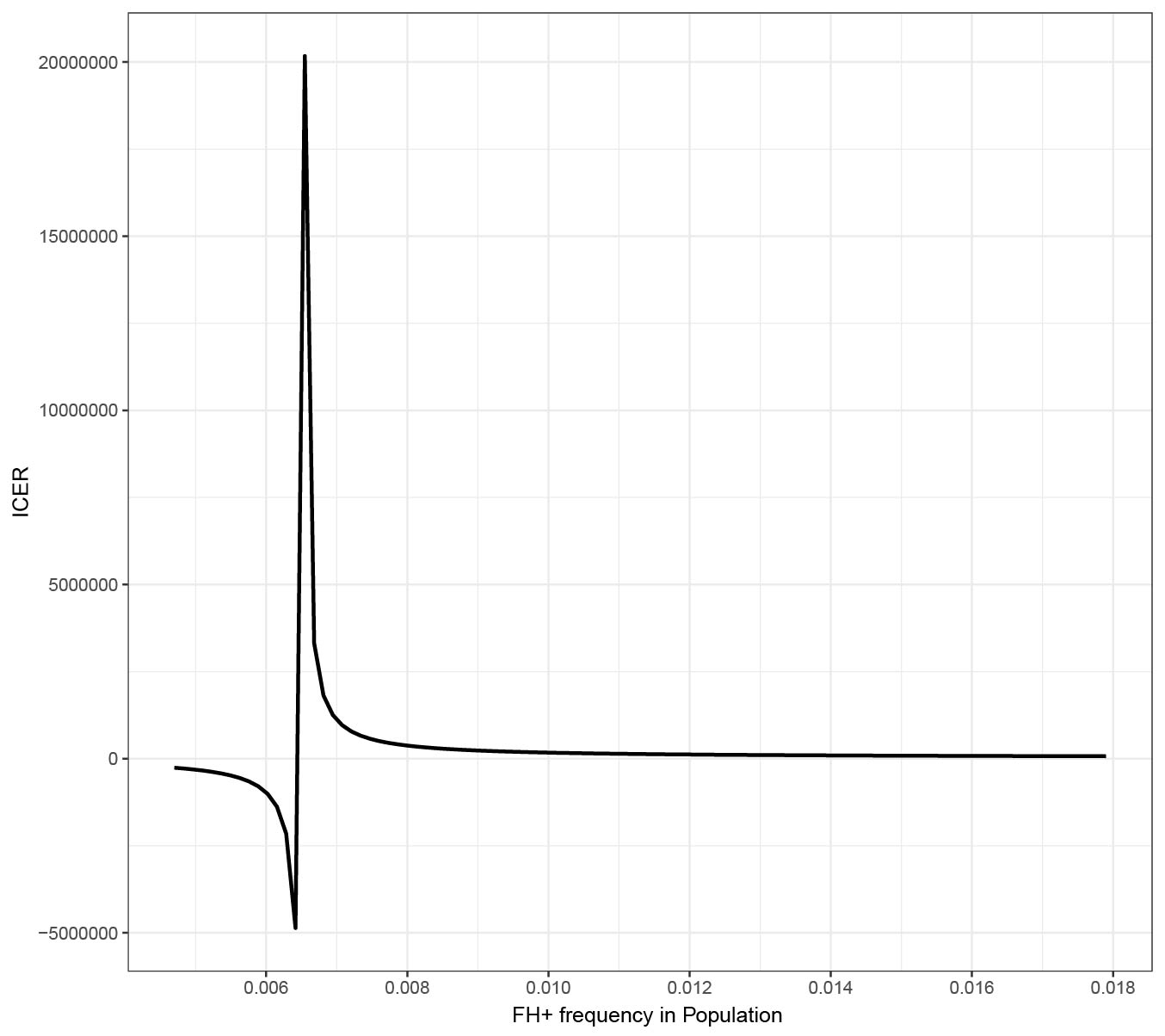

Supplement: SUPPLEMENTARY FIGURE 2 — ICER of FH-based strategy versus Symptom-only varies with changes in the family history positivity rates of breast and ovarian cancer. As x approaches 0.0065, y approaches infinity. [file Image_2.jpeg]
